# Gut microbial communities of hybridising pygmy angelfishes reflect species boundaries

**DOI:** 10.1038/s42003-023-04919-7

**Published:** 2023-05-18

**Authors:** Megan J. Huggett, Jean-Paul A. Hobbs, Federico Vitelli, Michael Stat, Tane H. Sinclair-Taylor, Michael Bunce, Joseph D. DiBattista

**Affiliations:** 1 School of Environmental and Life Sciences, University of Newcastle, Ourimbah, NSW 2258 Australia; 2 Centre for Marine Ecosystems Research, School of Science, Edith Cowan University, 270 Joondalup Drive, Joondalup, WA Australia; 3 School of Biological Sciences, The University of Queensland, Brisbane, QLD 4069 Australia; 4 Trace and Environmental DNA (TrEnD) Laboratory, School of Molecular and Life Sciences, Curtin University, Perth, WA 6102 Australia; 5 Red Sea Research Center, Division of Biological and Environmental Sciences and Engineering, King Abdullah University of Science and Technology, Thuwal, 23955-6900 Saudi Arabia; 6 Australian Institute of Marine Sciences, Townsville, QLD Australia; 7 Institute of Environmental Science and Research (ESR), Kenepuru, Porirua, 5022 New Zealand; 8 Australian Museum Research Institute, Australian Museum, 1 William St, Sydney, NSW 2010 Australia

**Keywords:** Ecosystem ecology, Water microbiology, Microbiome

## Abstract

Hybridisation and introgression of eukaryotic genomes can generate new species or subsume existing ones, with direct and indirect consequences for biodiversity. An understudied component of these evolutionary forces is their potentially rapid effect on host gut microbiomes, and whether these pliable microcosms may serve as early biological indicators of speciation. We address this hypothesis in a field study of angelfishes (genus *Centropyge*), which have one of the highest prevalence of hybridisation within coral reef fish. In our study region of the Eastern Indian Ocean, the parent fish species and their hybrids cohabit and display no differences in their diet, behaviour, and reproduction, often interbreeding in mixed harems. Despite this ecological overlap, we show that microbiomes of the parent species are significantly different from each other in form and function based on total community composition, supporting the division of parents into distinct species, despite the confounding effects of introgression acting to homogenize parent species identity at other molecular markers. The microbiome of hybrid individuals, on the other hand, are not significantly different to each of the parents, instead harbouring an intermediate community composition. These findings suggest that shifts in gut microbiomes may be an early indicator of speciation in hybridising species.

## Abstract (web summary)

Gut microbiomes of parent species of pygmy angelfishes were different from each other while hybrid individuals showed low differences to parents, suggesting shifts in gut microbiomes as early indicators of speciation in hybridising species.

## Introduction

Hybridisation is widespread among plants and animals and instances of hybridisation are expected to increase with environmental change^1^. Hybridisation is of evolutionary importance because it can facilitate the transfer of genetic material between species, a process known as introgression^2^. In some cases, this transfer has no evolutionary consequences, while in other cases it can have major implications including the loss of species where genome-wide admixture results in two species collapsing into one^3,4^. Introgression can also provide an evolutionary lifeline in the form of genetic variation and adaptive potential^5^, and ultimately generate new species^6,7^. Some historical hybrid events have even produced key evolutionary innovations that result in the radiation of species, particularly where innovations enable hybrids to exploit novel niches^5,8,9^.

An understudied component of hybridisation and introgression is the effect of these events on host gut microbiomes, which enable the digestion of dietary items but also interact with the host immune system^10^. Commensal gut bacteria may have co-evolved with their host animals^11–13^ given that evolutionary change in gut bacteria can occur rapidly (~15–18 generations in guppies^14^). A framework for understanding the evolution of complex communities of host organisms and their associated microbes has been proposed, including the definition of these complex entities as a single holobiont^15^ and the related idea that evolution acts upon these complex communities via the hologenome. Hologenomes are considered single evolutionary units that exhibit collaborative phenotypes cooperatively subject to evolutionary forces^16,17^. With regards to fish holobionts, the gut microbiome likely reflects selection pressures acting at the host and microbial level, but these may be mismatched given the faster turnover and shorter generation times of the latter versus the former. The gut microbiome may consequently serve as an early ‘biological’ indicator of speciation via hybridisation, a hypothesis that we here investigate in coral reef fishes.

Coral reef fishes represent the most species rich vertebrate communities in the world. Hybridisation is common in these fishes and we focus our study on angelfishes (family Pomacanthidae), which have the highest proportion of recorded hybrids for any coral reef fish family^18,19^. Hybridisation is particularly common among pygmy angelfishes (genus *Centropyge*), which appear to have recently diverged and are hybridising in narrow regions of secondary contact^18,20,21^. A well-studied case of angelfish hybridisation is that between the Lemonpeel Angelfish *Centropyge flavissima* and Eibli’s Angelfish *Centropyge eibli*. The geographic ranges of these species are largely separate, except for a region of overlap at Christmas Island in the Eastern Indian Ocean where hybridisation occurs^20,22^ with estimated divergence times between mitochondrial lineages of 3.5 to 4.2 million years^21,23,24^.

**Fig. 1:**
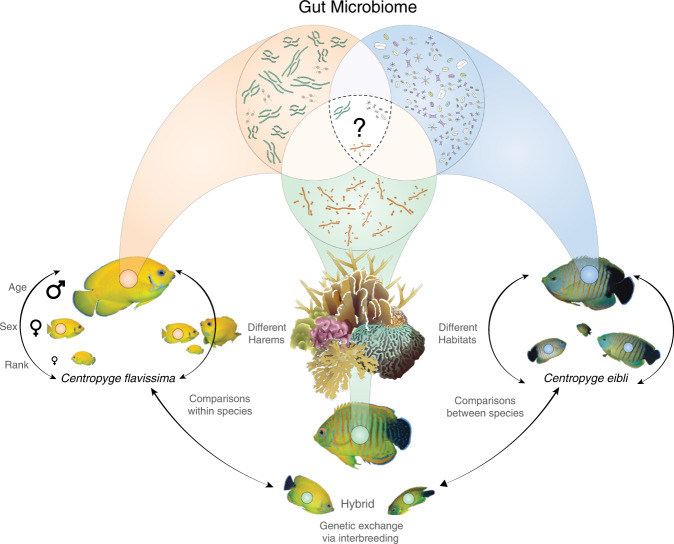
Overview of factors that may impact gastrointestinal microbial communities of hybridising reef fishes. Conceptual diagram of the various factors that may influence the microbial community composition of hybridising reef fishes such as pygmy angelfishes. These include factors such as age and sex of individuals, their position within a harem, the specific harem that they belong to, and the makeup of individuals with a harem, use of the surrounding habitat, and the extent of genetic exchange via interbreeding. Photos of fish taken by Tane Sinclair-Taylor (co-author), all other images sourced from the Symbols and Integration and Application Network (ian.umces.edu/media-library).

In the zone of overlap, parent species and their hybrids cohabit^25,26^ and display no differences in their preferred diet of predominately red, brown, and green algae^26^. The behaviour and reproduction of these hybridising species are also similar as they form mixed species harems comprised of one large male and three to six small females that occupy a small territory^25,27^. Due to similarity in habitat and food resources, territories often overlap^25^, but mating only occurs between members of the same harem^27^. Therefore, despite clear differences in individual body colour, mixed-species harems are common and stable, and deliberate interbreeding occurs within these harems; hybrid offspring breed with parentals once fully mature^27^. This extensive hybridisation leads to widespread introgression, with mitochondrial and nuclear markers showing no delineation between species^21,23,28,29^. Thus, these recently diverged species appear to overcome pre- or post-zygotic barriers to reproductive isolation, which means we can here address our hypothesis that microbiomes act as early indicators of speciation in hybridising animals (see Fig. 1).

## Results

### Selection of gut section

**Fig. 2:**
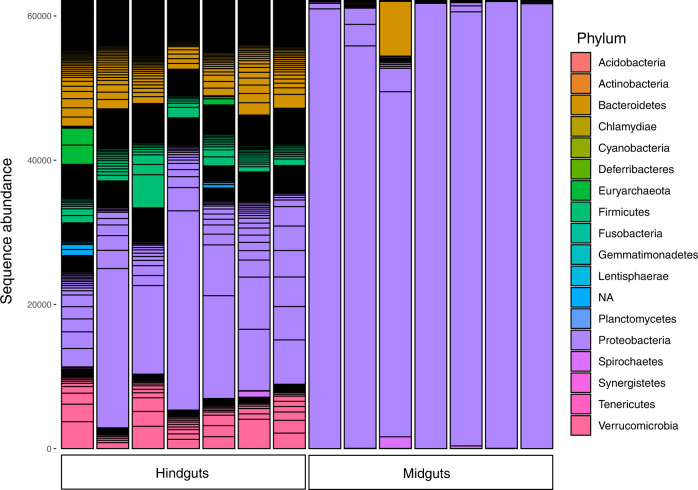
Abundance of prokaryotic phyla within the mid and hindguts of the lemonpeel angelfish *Centropyge flavissima* (*n* = 7). Total sequence abundance of phyla in midguts and hindguts of *Centropyge flavissima* (*n* = 7). Black horizontal bars indicate individual ASVs within phyla. HG hindgut sample, MG midgut sample.

Initially, microbial composition of the midgut and hindgut of *Centropyge flavissima* were compared to select the most appropriate region for comparison among species and hybrids. *Centropyge flavissima* midgut samples (*n* = 7) generated a mean of 88,042 sequences (SD ± 19,116) and 86 ± 60 OTUs per fish (total of 405 OTUs). Midguts were dominated by OTU1 (77–99% of sequence abundance, Fig. 2, Supplementary Data 1), which was most similar (98.2% match) to an *Endozoicomonas* sequence from coral tissue of *Porites lobata* archived in the National Center for Biotechnology Information (NCBI) database (GenBank accession number: KF180123). Of the seven individuals examined, five hosted >96.8% abundances of OTU1. For the remaining two individuals, one contained an additional 8.3% combined sequence abundance of OTU2 and OTU26, both of which also most closely matched to *Endozoicomonas* sequences (Table S1).

Paired hindgut samples from the same individuals generated a mean of 79,515 sequences (SD ± 12,695) and 1030 ± 82 OTUs per fish (total of 1402 OTUs). Hindgut samples also contained high abundances of OTU1 (mean abundance 20.9 ± 15.2%, Fig. 2, Table S1) but contained other abundant OTUs that were most similar to those from the hindguts of fish and other vertebrates including OTU5, most closely matched to a *Victivallales* sequence from the hindgut of a surgeonfish (97.4% similarity, GenBank accession number: KT952825); OTU4, most closely matched to a *Desulfovibrio* sequence from the hindgut of an angelfish (98.5% similarity, GenBank accession number: EU885154); OTU3, most closely matched to a *Desulfovibrio* sequence from the hindgut of the long nosed bandicoot (97.8% similarity, GenBank accession number: AY554146); and OTU6, most closely matched to a *Desulfovibrio* sequence from the hindgut of a surgeonfish (96.7% similarity, GenBank accession number: EU885140).

All alpha diversity metrics (observed numbers of species, Chao1, Simpson Diversity Index, and Shannon Diversity Index) were higher in hindguts versus midguts (*p* < 0.01, Fig. S1, Table S2). Beta diversity, as measured by the Bray-Curtis index, also differed between midgut and hindgut samples (PERMANOVA, *p* < 0.01, Table S3), with midgut communities having higher beta dispersion (*p* < 0.01) in comparison to hindguts (Fig. S2).

Of the 388 functional traits identified using predictive metagenomic analysis (Meta-Cyc data), 195 were identified as differing between the midgut and hindgut samples (LDA > 2.0, Supplementary Data 2, Fig. S3). Of these, 130 were present in higher than predicted normalised copy number in hindgut samples, and the remaining 65 were higher in midgut samples (Supplementary Data 2). Those functions that were predicted as higher in hindguts were mostly related to fermentation, including fermentation of amino acids and anaerobic fermentation of purines; the biosynthesis of amino acids, including L-lysine, L-arginine and L-valine; and the biosynthesis of carbohydrates, including heptose sugars. The functions that were predicted as being higher in midguts were related to biosynthesis of vitamins, cofactors and nucleic acids and the degradation of diverse organic molecules (carbohydrates, nucleotides, fatty acids, and lipids).

### Hindgut microbial communities

**Fig. 3:**
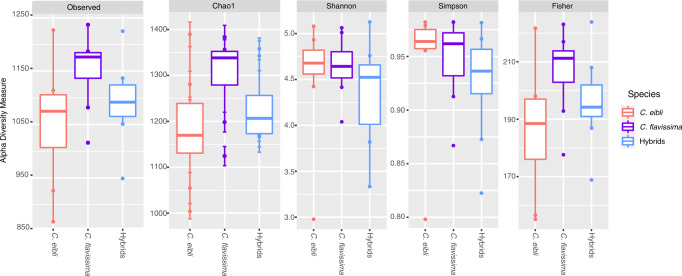
Alpha diversity of hindgut microbial communities of pygmy angelfishes and their hybrids (*n* = 8). Alpha diversity of hindgut microbial communities based on 16S rDNA gene sequencing. No differences were detected among sample types for any alpha diversity measures (one-way ANOVA, *p* < 0.05). Observed species number and chao index were calculated using rarefied data; Shannon, Simpson and Fisher’s indices were calculated using unrarefied data. *C. eibli* = *Centropyge eibli, C. flavissima* = *Centropyge flavissima*, Hybrids = hybrids of *C. eibli* and *C. flavissima* (*n* = 8). Boxes denote the interquartile range between the 25th and 75th percentiles of the data, and the horizontal line inside the box reflects the median value. The upper and lower whiskers represent scores outside this middle 50%. Dots outside whiskers represent outliers.

For comparisons between parent species and hybrids, the hindgut was preferred over the midgut as it provided a better representation of species rich, resident bacteria in fish, and sequences from hindguts were dominated by bacteria that were most closely aligned to sequences in NCBI from the gastro-intestinal tracts of fish and other vertebrates. In contrast, midguts were dominated by sequences most closely aligned to sequences in NCBI that were from the external environment, rather than from gastro-intestinal tracts of vertebrates, in particular to coral-associated and sponge-associated sequences (Table S1, Figs. S1 and S2). *Centropyge flavissima, C. eibli*, and hybrid hindgut samples from fish of the same length (1 way ANOVA, *p* > 0.05, Table S4, *n*  = 8 of each species) were examined. These generated a mean of 81,109 (SD ± 11,740), 81,909 (SD ± 12,189) and 77,048 (SD ± 16,464) sequences and a total of 2456, 2472 and 2409 OTUs, respectively (Supplementary Data 1). Abundant OTUs were similar among sample types, and there were no differences in alpha diversity between hindgut sample types (*p* > 0.05, Table S5, Fig. 3).

**Fig. 4:**
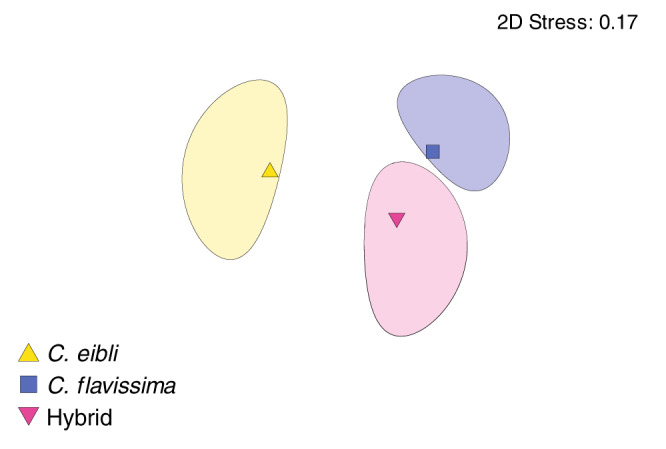
Non-metric multi-dimensional scaling (nMDs) plot comparing hindgut microbial community composition of pygmy angelfishes and their hybrids (*n* = 8). Two-dimensional nMDS ordination plot and 80% confidence ellipses constructed from bootstrap averages for the Bray-Curtis similarity index of square root transformed hindgut microbial community data from the two angelfish species, *Centropyge flavissima* and *C. eibli*, and their hybrids (*n* = 8).

**Fig. 5:**
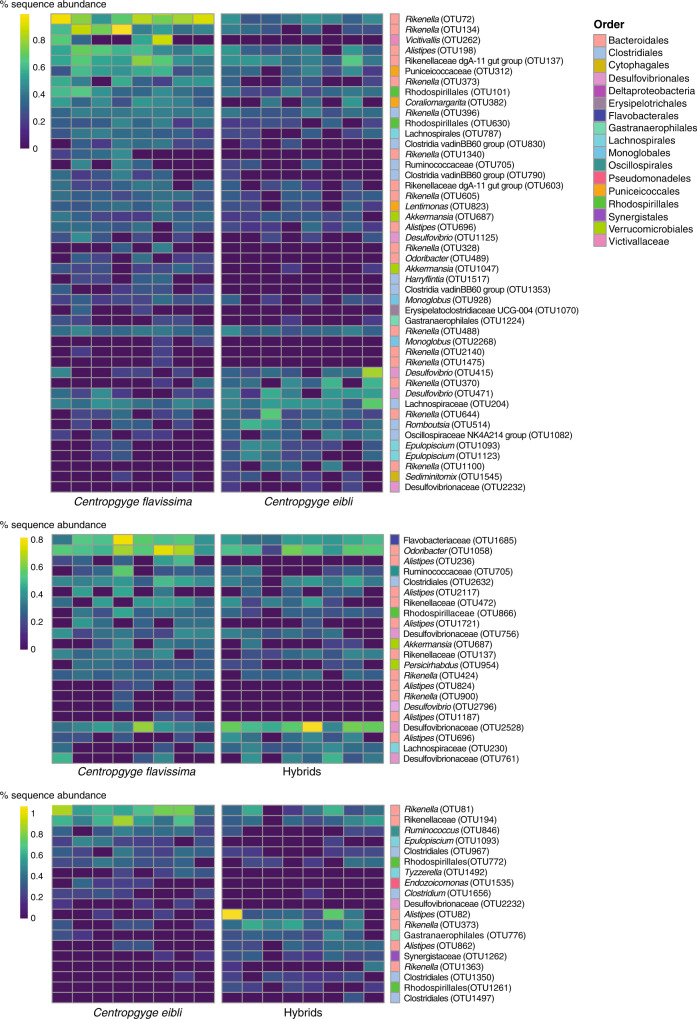
Abundance of prokaryotic operational taxonomic units (OTUs) that differ within hindguts of the pygmy angelfish and their hybrids (*n* = 8). Percent sequence abundance of OTUs that were significantly different within hindguts of the two angelfish species, *Centropyge flavissima* and *C. eibli*, and their hybrids (*n* = 8) based on mvabund analysis (*p* < 0.01). Taxonomic ranks are listed to genus, or the lowest possible taxonomic rank otherwise.

Despite these similarities, PERMANOVA analysis based on the Bray-Curtis Index indicated that there was a significant difference in the overall hindgut microbial community composition among sample types, with post-hoc tests indicating that samples from parent species were significantly different from each other, but the hybrids were not significantly different from either parent species (*p* < 0.05, Fig. 4, Table S6). Forty six OTUs differed in abundance between parent species and these were dominated by anaerobic fermenters, typical of gut microbiomes, including members of the Bacteroidetes genera *Rikenella* and *Alistipes*, the Proteobacteria genera *Desulfovibrio*, and members of *Firmicutes*. There were fewer OTUS that differed in abundance between hybrids and parent species; 22 OTUs were statistically different between hybrids and *C. flavissima*, and only 19 were statistically different hybrids and *C. eibli* (mvabund, p < 0.05, see Fig. 5). There were no statistical differences in hindgut microbial communities between sex, social rank or age across the parent species and hybrids (PERMANOVA, *p* > 0.05, Tables S7-S9). Within *Centropyge flavissima* (*n* = 17) there were also no statistical differences in overall microbial community composition of the hindguts across sex, social rank or age (PERMANOVA, *p* > 0.05, Tables S10–12).

Seventeen functional traits were identified as significantly different between the two parents species or hybrid hindgut samples (Supplementary Data 2, Fig. S4). Functions that were predicted as being higher in *C. flavissima* were related to the biosynthesis of vitamins including tetrahydrofolate as well as the fermentation and degradation of amino acids, whereas functions that were predicted as being higher in *C. eibli* were related to biosynthesis of the cofactor adenosylcobalamin and the amino acids L-phenylalanine and L-tyrosine, and the degradation of carbohydrates. The single function that was enriched in hybrids was related to the biosynthesis of L-methionine. Weighted nearest sequences taxon index (NSTI) scores averaged 0.34 ± 0.07 SD for hindgut samples and 0.19 ± 0.02 SD for midgut samples (Table S13). The NSTI is the sum of phylogenetic distances for each OTU within the dataset to its nearest relative with a sequenced genome; the higher the NTSI score the less accuracy there is in the metagenome predictions^30^. Midgut NSTI was within the range of environmental samples that have previously been accurately predicted^30^, but hindguts were much higher, suggesting that the predicted metagenomes of these samples are less reliable.

## Discussion

Our analysis of the gut microbial communities of two angelfish species and their hybrid offspring produced two noteworthy results. Firstly, despite sharing the same habitat^25^, diet^26^, harems^27^ and being indistinguishable at a number of phylogenetic marker genes^29^, the hindgut microbial communities of the parent species *C. flavissima* and *C. eibli* were statistically different to one another at the entire community level, and based on the identified core microbial community. However, the microbial communities of hybrids were not statistically different to either parent. This finding provides the only evidence, other than colouration, that *C. flavissima* and *C. eibli* indeed represent distinct species. Secondly, we found an unusually low species diversity within the midgut section of *C. flavissima*, with up to 99% relative abundance of a single OTU, identified as an *Endozoicomonas*, which dominated (>96% relative abundance) the midguts of the majority of individuals. This discovery may represent an important symbiosis within the gut of *C. flavissima* angelfish.

Within the marine environment, coral holobionts have been most widely studied and while the holobiont concept has been generally accepted the hologenome concept is a hypothesis subject to ongoing debate^31–34^. The hologenome concept theorises that natural selection acts upon the holobiont to increase the fitness of the holobiont overall regardless of fitness of the individual units within the holobiont^16,17^. This idea is potentially problematic since many microbes that comprise complex holobionts do not live strictly within a single host and may associate with other environments or only with specific life stages of a host, suggesting their evolution will be both linked to, and independent of, the holobiont. A more moderate version of the hologenome concept hypothesises that evolution acts on individual members within the holobiont differentially and is influenced by both host and microbial genetic factors, that competition within the microbial community is influenced by microbial genetic variation, and that there is a strong role for environmental drivers of microbial variation within holobionts^31^. The evidence presented here provides support for this moderate view of the hologenome, indicating that evolution of microbial communities occurs more rapidly than host fish but that there is co-evolution of microbes with their fish hosts. Furthermore, we provide evidence for host genotype shaping the microbial community, since hybrids are host to a microbial community that contains a mix of each parent species as well as a subset of unique taxa, possibly an early indicator of speciation via hybridisation.

Although host microbiome co-evolution in fish has not been explicitly tested, some fish microbiomes appear consistent with population genetic divergence (salmonids^35^) and local adaptation to novel environments (rabbitfish^36^). A recent study has also demonstrated that wild salmon gut microbial communities are dominated by *Mycoplasma* clades, which vary subtly among individuals and follow expected patterns of co-evolution across host species^13^. Interestingly, while the skin microbiome of elasmobranchs follows a pattern of co-evolution and phylosymbiosis, the skin microbiome of teleost fishes lack a consistent phylosymbiosis relationship^37^. The specific role of hybridisation and introgression of parental fish species in determining fish microbiome composition has been investigated in laboratory^38^ and aquaculture settings^39,40^, but is under-studied in wild populations. In a laboratory setting, intestinal microbial communities of captive whitefish were found to reflect parental or hybrid origin, with overall community composition as well as particular phyla and genera being distinct among parents and F1 hybrids. However, the intestinal communities in the laboratory setting were altered when compared to those from the wild^38^. In a slightly more natural setting of aquaculture pens, intestinal microbial communities of hybrid carp were transitional between parental species, with hybrids hosting some specific taxa in their foreguts that suggest the beginnings of a unique niche of the gut microbiome of hybrids^40^. Given that the gut microbial community structure can be significantly shaped by taxon, genetic background, and feeding habit^41,42^, our results are noteworthy because they demonstrate variation in microbiome composition between species and their first generation progeny in a natural environment, where a number of host-associated factors (e.g. location, diet, habitat) are as similar as possible in a wild setting.

The differences between species in gut microbiomes is surprising given that their ecological similarities should expose them to the same microbial community. Based on sharing the same habitat^25^, diet^26^, social groups and interbreeding^27^, we expected the microbial community composition to be similar between all hindgut samples collected from these fish. The differences in microbiomes indicates selection may be occurring for different microbial communities. Thus, we posit that the hindgut microbiome may serve as an indication of speciation at ecological time scales where mitochondrial and nuclear markers are indistinguishable^23,29^. Differentiation in microbiomes between all three groups of fish may reflect faster evolution of the microbial communities, or tight evolutionary constraints linked to the host species. Gut microbiomes may therefore act as a signature of the evolutionary trajectory of hybridisation, flagging the emergence of new species and subsequent increases in biodiversity.

The midgut of *C. flavissima* resembles a monoculture, with up to 99% relative abundance of a single OTU (most individuals host >96% relative abundance) that was identified as most closely related to sequences from the genus *Endozoicomonas*. This OTU was not detected in the procedural controls included in our work, providing convincing evidence that this is a true association within this region of the gut. Members of the *Endozoicomonas* are facultative anaerobes^43^ and there is growing evidence for their importance as symbionts in marine organisms including corals^44^, sponges^45^ and ascidians^46^. A recent whole genome analysis of a sponge-associated *Endozoicomas* strain identified eukaryote-like proteins thought to promote attachment to the sponge host, suggesting some *Endozoicomonas* strains are well adapted to a symbiotic lifestyle^45^. *Endozoicomonas* are abundant in both shallow^44,47,48^ and deep-sea corals^49^ in some cases forming abundances as high as seen here. Obligate corallivore species of butterflyfish can also host high abundances of *Endozoicomonas* within their intestinal microbial communities (up to about 75% relative sequence abundance), dependant on reef location^50^. The monoculture-like growth of this strain in the midgut suggests that a strong selection pressure exists for this association and a possible symbiosis, similar to patterns observed for some individuals within wild Atlantic salmon populations that host abundances of up to 90% sequence abundance of the Tenericutes genus *Mycoplasma*^35^. Coral-associated *Endozoicomonas* may be acquired horizontally either incidentally or via diet; and for corallivores such as butterflyfishes these strains may aid in the digestion of coral tissues^50^. However, the angelfish examined here are herbivorous, with a diet consisting of brown and red macroalgae^26^, and the functional role of this strain in the gut requires further investigation. Low abundances of *Endozoicomonas* have also been identified in other reef dwelling fish (e.g. damselfish and cardinalfish^51^; rabbitfishes^36^; humphead Māori wrasse flagtail rockcod, bluestriped snapper and goldspot seabream^52^), and *Endozoicomonas* are enriched in gill-associated microbial communities of reef fishes^53^. Such a perennial association suggests that reef fishes may play an ecological role as a vector for inoculation of key members of other holobionts including corals and sponges.

When microbial communities differ, as we found in this study, it does not necessarily result in functional changes since different microbial taxa are capable of comparable functional roles (i.e., functional redundancy^54^). However, based on Linear Discriminant Analysis (LDA) Effect Size (LEfSe)^55^ analysis of predicted functional genes, our results indicate several functional traits are predicted to differ between parent species. However, comparative metagenome and functional data are lacking for wild fish gut microbial communities to fully explore the importance of this observed distinction. For example, a recent metagenomic study of deep sea fish uncovered 111 assembled metagenomes and only 39 of these had >75% average nucleotide similarity (ANI) to publicly available genomes, indicating a lack of genomic information from this environment^56^. This paucity in genome coverage is also reflected in the high ANI scores returned from our PiCRUST analysis. Here, hybridisation appears to have resulted in a mixing of parental microbial communities, which may allow hybrids to eventually form distinct microbial associations when compared to either parent. In this case, the functional repertoire of the hybrid microbial community will likely play an important role in determining whether hybrids will in turn be able to exploit a unique niche from their parents, possibly facilitating reproductive isolation and eventually speciation.

Hybridisation and introgression are powerful evolutionary forces, and here we show that their effects on host gut microbiomes in natural populations of coral reef fishes may provide another layer in the process of speciation. Indeed, the variable and the more conserved fractions of the gastrointestinal microbial community were different among parent fish species but intermediate in their hybrids, which form reproductively active and behaviourally incorporated members of their haremic mating system. We observed this differentiation in gut microbiomes between parental species and hybrids despite remarkable similarity in habitat use, diet, and genetic markers aimed at delineating between them. These findings suggest that shifts in gut microbiomes may be an early indicator of speciation in hybridising species via mechanisms of co-evolution where microbial community turnover occurs at a faster rate than the host.

## Methods

### Study sites and sample collection

Pygmy angelfish from two species (*Centropyge flavissima* and *C. eibli*) and their hybrids were collected in Flying Fish Cove (10°25′45.7′′S, 105°40′05.7′′E) at Christmas Island in September 2015, a known hybrid hotspot in the eastern Indian Ocean (Hobbs et al. 2009, 2014)^22,57^. For each individual that we collected, we recorded the location and composition of their harem, total length (TL) was measured to the nearest 1 mm, and the two sagittal otoliths were removed and stored dry. Gonads were removed and preserved in a fixative containing 4% formaldehyde, 5% acetic acid and 1.3% calcium chloride for 24 h, then transferred to ethanol. The mixed species harems that we observed have remained stable (same location on the reef and identical harem members) for between 1 and 6 years. Hybrids were identified in situ based on intermediate body colouration. Whole fish specimens were collected by hand spear, immediately placed on ice, and processed within four hours of collection. Fish samples were collected under fisheries exemption permit number 2087 issued by the Department of Fisheries WA, and ethically approved (AEC_2015_25) by the Animal Ethics Committee at Curtin University (Australia). The permit and ethics approvals were issued to J.P.H.

The entire gastrointestinal (GI) system was dissected out of fishes; individuals with damaged guts were excluded. The gut morphology of the different species and their hybrids were indistinguishable, with all individuals having a large terminal sac (referred to herein as the hindgut). Samples were taken from the hindgut of 17*C. flavissima* individuals and from the midguts of a subset of seven individuals. A further eight *C. eibli* and hybrids were collected for hindgut analysis. Gut material (~1 g) was removed and collected into separate, aseptic 80% ethanol suspensions from directly behind the stomach (midgut samples) and from the terminal end of the hindgut (hindgut samples). Samples were immediately frozen at −20 °C for up to 14 days, then stored at −80 °C. Diet was evaluated in a separate study by examining the contents of the stomachs^25^. The harem of each individual was recorded and is reported in Table S14.

### Otolith preparation and reading

The age of each individual was determined by sectioning sagittal otoliths following Wakefield et al.^58,59^. A dissecting microscope was used to quantify age (in years) based on the number of opaque zones in the otolith sections. All sections were examined independently by two readers, without knowledge of fish size without any knowledge of the size of fish. The precision between readers was compared using age-bias plots and the Index of Average Percent Error (IAPE^58,60^). The final age for each fish was based on the agreement between two readers.

### Gonads histological examination and maturity estimates

Preserved gonads were embedded in paraffin wax, sectioned transversally at ~5 μm, mounted on glass slides and stained with Harris’s haemotoxylin and Young’s eosin– erythrosine^61^. During microscopic examination of gonads, maturity was assigned based on the presence of vitellogenic oocytes in females, and spermatozoa in males. Further details of age, sex and rank for all individuals are provided in Table S15.

### DNA extraction, amplification and sequencing

Approximately 0.5 g of homogenised hindgut contents from each specimen were used to extract total genomic DNA using the PowerSoil Kit (Mo Bio Laboratories, Carlsbad, CA, USA) according to the manufacturers’ protocol. Sequencing from paired midgut and hindgut (n = 7 fish) extractions from *C. flavissima* and hindgut samples for *C. flavissima, C. eibli*, as well as their hybrids (*n* = 8 per species/hybrids, Supplementary Data 1) was performed on an Illumina MiSeq platform (Illumina, San Deigo, CA, USA) in the Trace and Environmental DNA (TrEnD) laboratory at Curtin University in Perth, Australia using 515F (5′ GTGBCAGCMGCCGCGGTAA 3′) and 806 R (5′ GGACTACHVGGGTAWTCTAAT 3′) primers^62^ targeting the V4 region of the 16S rDNA gene. The optimal yield of DNA for PCR reactions was first determined by qPCR^63^, then amplicons were generated using a single round of PCR with fusion tag primers including Illumina adapter regions, unique MID tags, and primers. Quantitative PCR (qPCR) experiments were performed in duplicate on a StepOnePlus Real-Time PCR System (Applied Biosystems, CA, USA) using 1× AmpliTaq Gold® Buffer, 2 mM MgCl_2_, 0.25 µM dNTPs, 10 ug BSA, 1U AmpliTaq Gold DNA polymerase, 2 µl template DNA and ultrapure distilled water to 25 µl. These reactions were amplified under the following conditions: initial denaturation at 95 °C for 5 min, then 35 cycles of 95 °C for 30 s, 50 °C for 30 s, and 72 °C for 45 s, and a final extension at 72 °C for 10 min. PCR duplicates of each sample were combined and all samples were then pooled in equimolar ratios. Extraction controls and PCR negative controls containing no sample were added to sequencing runs to assess for cross-contamination. Final sequencing libraries were size selected using a Pippin Prep (Sage Science, Beverly, MA, United States), purified using a QIAquick PCR purification Kit (QIAGEN, Venlo, Netherlands), and sequenced unidirectionally using an Illumina 300 cycle MiSeq v2 Reagent Kit and standard flow cell.

Sequence data was processed using MOTHUR v1.35.1^64^ following the MiSeq standard protocol^65^. Sequences <250 bp in length or with ambiguous base calls were removed and duplicates were merged, and aligned to the SILVA reference alignment. Chimeras were removed using vsearch and poorly aligned sequences with the filter.seqs command, both within the MOTHUR environment. Unique sequences were identified, the dataset was rarefied to the lowest number of sequences per sample (62,127 for comparisons between midguts and hindguts of *C. flavissima*, 41,967 for comparisons across hindgut samples of parents and hybrids), and operational taxonomic units (OTUs) (>97% similarity) were defined and classified against the SILVA v132 database. OTUs were favoured over ASVs in order to avoid inflation of species diversity due to heterogeneity of the 16S rDNA gene^66^. Any sequences classified as mitochondrial, chloroplast, eukaryotic, or of unknown origin were removed using MOTHUR.

### Statistics and reproducibility

Statistical analyses were conducted using R Studio (2021.09.1, build 372)^67^ and PRIMER v7^68^. Microbial community compositional differences between (i) hindgut and midgut samples of *C. flavissima* and (ii) hindgut samples of *C. flavissima, C. eibli*, and their hybrids were identified using model-based multivariate analysis (mvabund) with the *mvabund* package^69^ as implemented in R Studio. The comparison of parents and hybrids spans individuals from 12 harems. Across the dataset, there were no harems where both parent species and hybrids were examined. That is, it is unlikely that we have directly sampled pairs of parents and their direct offspring, but instead have examined a haphazard selection of individuals from each species and the hybrids. Community composition of hindgut samples was visualised in PRIMER v7 with a two-dimensional nMDS ordination plot and 80% confidence ellipses constructed from bootstrap averages for Bray-Curtis similarity of square root transformed data. *Mvabund* was also used to identify individual OTUs that differed between sample types and these differences were visualised as heatmaps using the R package *superheat*^70^. Analysis of multivariate homogeneity of group dispersions (variances) was done using the R package *Vegan*^71^. The R package *phyloseq*^72^ was used to visualise alpha diversity using boxplots, with means of these and total fish length (mm) each compared using one-way analysis of variance (ANOVA) followed by Tukey’s post-hoc tests using the R packages *tidyverse*^73^ and *AICcmodavg*^74^. Data were checked to ensure that they fit the assumptions of ANOVA and square-root transformed where necessary. Core microbiome OTUs (those present in 100% of samples within either a parent or hybrid sample set) were identified. PICRUSt2 (Phylogenetic Investigation of Communities by Reconstruction of Unobserved States 2) pipeline version 2.3.0-b^75^ with default parameters was used to generate predicted functional profiles of microbial communities based on 16S rRNA marker genes. PICRUSt2 predicts the functional capabilities of the 16S rRNA sequencing data by phylogenetically comparing these to all publicly available whole genomes for bacteria and archaea and estimating genomic copy numbers of each gene family based on phylogenetic similarity. During this process, OTUs were normalised by copy number and a weighted nearest sequences taxon index (NSTI) score was calculated, with those above 2.0 removed from the analysis. Enzyme commission (EC) metagenomes and MetaCyc pathway abundances were inferred, and similarities between the overall functional profiles of samples were visualised using Statistical Analysis of Metagenomic Profiles^76^ as principal components analysis plots. The galaxy online portal was used to compare MetaCyc pathways that were differentially abundant between sample types using the LDA LEfSe method^55^ following normalisation (https://huttenhower.sph.harvard.edu/galaxy/).

### Reporting summary

Further information on research design is available in the Nature Portfolio Reporting Summary linked to this article.

## Supplementary information

Supplementary Information

Description of Additional Supplementary Files

Supplementary Data 1

Supplementary Data 2

Reporting Summary

## Data Availability

The genetic data are publicly available in the Sequence Read Archive of the NCBI sequence database under BioProject PRJNA878543 with accession numbers SAMN30732737 to SAMN30732771.
