# Supplementary material for: Gut microbial communities of hybridising pygmy angelfishes reflect species boundaries
Source: Commun Biol. 2023 May 18;6:542. doi: 10.1038/s42003-023-04919-7 (PMC10195815; doi:10.1038/s42003-023-04919-7)

Supplementary Information

Table S1: Closest sequence matches in NCBI to abundant OTUs from midgut (MG) and hindgut (HG) sections of *Centropyge flavissima* (n=7). GI: gastrointestinal tract.

**OTU Number**	**Environment/Host of most similar sequence**	**Assigned Identification**	**Accession number**	**Match (% similarity)**	
**OTU1**	Marine coral *Porites lutea* healthy tissue	*Endozoicomonas*	KF180123	98.17	Abundant in MG and HG
**OTU2**	Marine coral *Stylophora pistillata*	*Endozoicomonas*	KR233752	98.53	Abundant in MG and HG
**OTU26**	Marine coral *Porites lutea* healthy tissue	*Endozoicomonas*	KF180123	98.17	Abundant in MG
**OTU40**	Marine sponge *Axinella verrucosa*	*Aureibacter*	JN699196	96.30	Abundant in MG
**OTU87**	Marine coral *Acropora millepora*	*Ferrimonas senticii*	MW828531	99.27	Abundant in MG
**OTU150**	Marine herbivorous fish *Naso unicornis* GI	*Brevinema*	HM630215	95.60	Abundant in MG
**OTU3**	Long nosed bandicoot lower bowel	*Desulfovibrio*	AY554146	97.81	Abundant in HG
**OTU4**	Marine angelfish *Pomacanthus sexstriatus* GI	*Desulfovibrio*	EU885154	98.53	Abundant in HG
**OTU5**	Marine herbivorous fish *Naso hexacanthus* GI	Victivallales	KT952825	97.44	Abundant in HG
**OTU6**	*Pomacanthus sexstriatus* GI	*Desulfovibrio*	EU885140	96.7	Abundant in HG
**OTU7**	*Rangifer tarandus tarandus* (Norwegian reindeer) rumen	Oscillospiraceae	DQ394594	94.18	Abundant in HG
**OTU8**	Marine angelfish *Pomacanthus sexstriatus* GI	Lentisphaeria	EU885062	98.9	Abundant in HG
**OTU9**	Marine angelfish *Pomacanthus sexstriatus* GI	*Desulfovibrio*	EU885122	98.53	Abundant in HG
**OTU10**	Marine angelfish *Pomacanthus sexstriatus* GI	*Desulfovibrio*	EU885150	98.17	Abundant in HG
**OTU11**	Marine angelfish *Pomacanthus sexstriatus* GI	*Akkermansia*	EU885079	96.7	Abundant in HG
**OTU12**	Marine herbivorous fish *Naso unicornis* GI	*Alistipes*	KT952838	95.97	Abundant in HG
**OTU13**	Marine angelfish *Pomacanthus sexstriatus* GI	*Desulfovibrio*	EU885153	98.9	Abundant in HG
**OTU14**	Marine angelfish *Pomacanthus sexstriatus* GI	Desulfovibrionaceae	EU885108	96.34	Abundant in HG
**OTU15**	Marine angelfish *Pomacanthus sexstriatus* GI	Lachnospiraceae	EU885016	98.17	Abundant in HG
**OTU16**	Marine angelfish *Pomacanthus sexstriatus* GI	*Akkermansia*	EU885079	96.34	Abundant in HG
**OTU17**	Marine angelfish *Pomacanthus sexstriatus* GI	*Desulfovibrio*	EU885104	95.97	Abundant in HG
**OTU18**	Marine herbivorous fish *Naso tonganus* GI	*Akkermansia*	HM630194	95.24	Abundant in HG
**OTU19**	Seawater around coral	*Alistipes*	KJ601326	94.51	Abundant in HG
**OTU20**	Marine angelfish *Pomacanthus sexstriatus* GI	Desulfovibrionaceae	EU885114	97.8	Abundant in HG
**OTU21**	Seawater around coral	*Alistipes*	KJ601326	94.51	Abundant in HG
**OTU22**	Marine angelfish *Pomacanthus sexstriatus* GI	Desulfovibrionaceae	EU885108	98.17	Abundant in HG
**OTU23**	Marine herbivorous fish *Zebrasoma desjardinii* GI	*Akkermansia*	KT952863	97.07	Abundant in HG
**OTU24**	Seawater around coral	*Alistipes*	KJ601326	94.14	Abundant in HG
**OTU25**	*Ctenopharyngodon idellus* (grass carp) GI	*Desulfovibrio*	JN032893	96.72	Abundant in HG

Table S2: ANOVA results of *Centropyge flavissima* hindgut vs midgut microbial community alpha diversity indices (n=7).

Observed species richness (Obs)

Source of variation	Df	Sum Sq	Mean Sq	F-value	P-value
**Gut section**	1	4182365	4182365	82.4	***3.78e-12***
**Residuals**	12	67926	5661		

Chao1

Source of variation	Df	Sum Sq	Mean Sq	F-value	P-value
**Gut section**	1	4540710	4540710	733	***3.97e-12***
**Residuals**	12	74336	5661		

Shannon Diversity Index

Source of variation	Df	Sum Sq	Mean Sq	F-value	P-value
**Gut section**	1	59.01	59.01	356.6	***2.72e-10***
**Residuals**	12	1.99	0.17		

Fisher's alpha parameter

Source of variation	Df	Sum Sq	Mean Sq	F-value	P-value
**Gut section**	1	122604	122604	736.3	***3.86e-12***
**Residuals**	12	1998	167		

Simpson Diversity Index

Source of variation	Df	Sum Sq	Mean Sq	F-value	P-value
**Gut section**	1	2.3545	2.3545	193.2	***9.26e-09***
**Residuals**	12	0.1462	0.0122		

Table S3: PERMANOVA results of *Centropyge flavissima* hindgut vs midgut microbial community based on Bray Curtis Similarity (n=7).

Source of variation	Df	Sum Sq	Mean Sq	Pseudo-F	P(perm)
**Gut section**	1	1.6773	1.67734	6.5464	***0.003***
**Residuals**	12	3.0747	0.25622		

Table S4: ANOVA results comparing total length (TL) of *Centropyge flavissima* (mean 86.6 ± 11.1 mm, n=10), *C. eibli* (mean 88.8 ± 6.2 mm, n=8) and hybrids (mean 89.3 ± 14.6 mm, n=8).

**Source of variation**	**Df**	**Sum Sq**	**Mean Sq**	**F-value**	**P-value**
**Species**	2	20.9	10.46	0.084	0.919
**Residuals**	25	3104.0	124.16		

Table S5: ANOVA results comparing *Centropyge flavissima, C. eibli* and hybrids hindgut microbial community alpha diversity indices (n=8).

Observed species richness (Obs)

Source of variation	Df	Sum Sq	Mean Sq	F-value	P-value
**Fish species**	2	47065	23533	3.011	0.0708
**Residuals**	21	164102	7814		

Chao1

Source of variation	Df	Sum Sq	Mean Sq	F-value	P-value
**Fish species**	2	50126	25063	3.066	0.0679
**Residuals**	21	171664	8174		

Shannon Diversity Index

Source of variation	Df	Sum Sq	Mean Sq	F-value	P-value
**Fish species**	2	0.339	0.1694	0.586	0.565
**Residuals**	21	6.068	0.2889		

Fisher's alpha parameter

Source of variation	Df	Sum Sq	Mean Sq	F-value	P-value
**Fish species**	2	1688	844.0	2.665	0.093
**Residuals**	21	6650	316.7		

Simpson Diversity Index

Source of variation	Df	Sum Sq	Mean Sq	F-value	P-value
**Fish species**	2	0.00257	0.0013	0.479	0.626
**Residuals**	21	0.05631	0.0027		

Table S6: PERMANOVA results of *Centropyge flavissima, C. eibli* and hybrids hindgut microbial community based on Bray Curtis Similarity (n=8).

Main test

**Source of variation**	**Df**	**Sum Sq**	**Mean Sq**	**Pseudo-F**	**P (perm)**
**Fish species**	2	2436.4	1218.20	1.3452	***0.015***
**Residuals**	21	19017.0	905.56		

Pairwise comparisons

**Comparison**	**t**	**P (perm)**
C. flavissima-C. eibli	1.2806	***0.022***
C. eibli-hybrids	1.0793	0.161
C. flavissima-hybrids	1.1131	0.056

Table S7: PERMANOVA results of comparison of sex among *Centropyge flavissima, C. eibli* and hybrid hindgut microbial community based on Bray-Curtis similarity index (n=13 males, n=11 females).

Source of variation	Df	Sum Sq	Mean Sq	Pseudo-F	P (perm)
**Sex**	1	0.207	0.054	1.261	0.2
**Residuals**	22	3.642	0.946		

Table S8: PERMANOVA results of comparison of rank among *Centropyge flavissima, C. eibli* and hybrid hindgut microbial community based on Bray-Curtis similarity index(n=11 rank 1, n=4 rank 2, n=3 rank 3 and n=4 rank 4). Rank 5 (n=2) excluded due to low sample size.

Source of variation	Df	Sum Sq	Mean Sq	Pseudo-F	P (perm)
**Sex**	3	0.450	0.128	0.882	0.685
**Residuals**	18	3.064	0.872		

Table S9: PERMANOVA results of comparison of age among *Centropyge flavissima, C. eibli* and hybrid hindgut microbial community based on Bray-Curtis similarity index (n= 5 age 3, n=2 age 4, n=7 age 5, n=2 age 6, n=2 age 7, n=2 age 8 and n=2 age 11). Ages 2 and 13 excluded due to low sample size (n=1).

Source of variation	Df	Sum Sq	Mean Sq	Pseudo-F	P (perm)
**Age**	1	0.233	0.064	1.388	0.135
**Residuals**	20	3.356	1.000		

Table S10: PERMANOVA results of comparison for sex within *Centropyge flavissima* hindgut microbial community based on Bray-Curtis similarity index (n=6 males, n=11 females).

Source of variation	Df	Sum Sq	Mean Sq	Pseudo-F	P (perm)
**Sex**	1	883.46	883.46	1.1351	0.167
**Residuals**	15	11675	778.34		

Table S11: PERMANOVA results of comparison for rank (rank 1 to 4) within *Centropyge flavissima* hindgut microbial community based on Bray-Curtis similarity index (n= 5 rank 1, n=2 rank 2, n=5 rank 3, n=4 rank 4). Rank 5 (n=1) excluded due to low sample size.

Source of variation	Df	Sum Sq	Mean Sq	Pseudo-F	P (perm)
**Sex**	3	0.412	0.193	0.956	0.509
**Residuals**	12	1.723	0.897		

Table S12: PERMANOVA results of comparison for age within *Centropyge flavissima* hindgut microbial community based on Bray-Curtis similarity index (n= 3 age 3, n=2 age 4, n=4 age 5, n=3 age 6, n=2 age 8). Ages 7, 11 and 13 excluded due to low sample size (n=1).

Source of variation	Df	Sum Sq	Mean Sq	Pseudo-F	P (perm)
**Age**	1	0.093	0.052	0.6675	0.788
**Residuals**	12	1.689	0.947		

Table S13: Weighted nearest sequences taxon index (NSTI) score of PICRUSt2 (Phylogenetic Investigation of Communities by Reconstruction of Unobserved States 2) outputs for samples in this study.

Sample ID	Gut section	Species	Weighted NSTI
**MG102**	Midgut	*Centropyge flavissima*	0.17954581
**MG107**	Midgut	*Centropyge flavissima*	0.172505758
**MG110**	Midgut	*Centropyge flavissima*	0.221772639
**MG120**	Midgut	*Centropyge flavissima*	0.182512915
**MG122**	Midgut	*Centropyge flavissima*	0.184939294
**MG123**	Midgut	*Centropyge flavissima*	0.183928669
**MG124**	Midgut	*Centropyge flavissima*	0.18269188
**HG1**	Hindgut	*Centropyge eibli*	0.317804454
**HG10**	Hindgut	*Centropyge eibli*	0.217294794
**HG45**	Hindgut	*Centropyge eibli*	0.304829948
**HG48**	Hindgut	*Centropyge eibli*	0.358999861
**HG51**	Hindgut	*Centropyge eibli*	0.314838955
**HG60**	Hindgut	*Centropyge eibli*	0.339915752
**HG76**	Hindgut	*Centropyge eibli*	0.38864729
**HG9**	Hindgut	*Centropyge eibli*	0.323498361
**HG101**	Hindgut	*Centropyge flavissima*	0.357638873
**HG102**	Hindgut	*Centropyge flavissima*	0.357367
**HG104**	Hindgut	*Centropyge flavissima*	0.345475412
**HG105**	Hindgut	*Centropyge flavissima*	0.350839516
**HG106**	Hindgut	*Centropyge flavissima*	0.353567586
**HG107**	Hindgut	*Centropyge flavissima*	0.332933454
**HG108**	Hindgut	*Centropyge flavissima*	0.317431472
**HG109**	Hindgut	*Centropyge flavissima*	0.326412644
**HG110**	Hindgut	*Centropyge flavissima*	0.382182733
**HG111**	Hindgut	*Centropyge flavissima*	0.378952344
**HG112**	Hindgut	*Centropyge flavissima*	0.414648854
**HG120**	Hindgut	*Centropyge flavissima*	0.336361654
**HG121**	Hindgut	*Centropyge flavissima*	0.348253465
**HG122**	Hindgut	*Centropyge flavissima*	0.335932053
**HG123**	Hindgut	*Centropyge flavissima*	0.359616171
**HG124**	Hindgut	*Centropyge flavissima*	0.336185591
**HG125**	Hindgut	*Centropyge flavissima*	0.364657439
**HG24**	Hindgut	*Centropyge eibli X Centropyge flavissima*	0.391931952
**HG28**	Hindgut	*Centropyge eibli X Centropyge flavissima*	0.372196432
**HG37**	Hindgut	*Centropyge eibli X Centropyge flavissima*	0.375988914
**HG67**	Hindgut	*Centropyge eibli X Centropyge flavissima*	0.33643966
**HG68**	Hindgut	*Centropyge eibli X Centropyge flavissima*	0.315967461
**HG83**	Hindgut	*Centropyge eibli X Centropyge flavissima*	0.273293133
**HG89**	Hindgut	*Centropyge eibli X Centropyge flavissima*	0.302298826
**HG95**	Hindgut	*Centropyge eibli X Centropyge flavissima*	0.374768905

Table S14: Distribution of hindgut microbial community samples of *Centropyge flavissima, C. eibli* and hybrids across harems

Harem	*C. eibli*	Hybrids	*C. flavissima*
A	1	0	0
B	1	0	0
C	0	1	0
D	0	0	2
E	0	1	0
F	0	1	0
G	0	0	2
H	0	2	2
I	4	1	0
J	1	0	3
K	0	1	0
L	1	1	0

Table S15: Details of social rank, total length, sex, maturity and age for individuals of each species and hybrid. Social rank was based on behavioural observations of social groups before collection and relative size (within each social group) of collected individuals. Sex and maturity were determined from microscopic examination of sectioned gonads. Females were classed as mature, or immature based on the presence or absence (respectively) of vitellogenic oocytes. Males were classed as mature, or immature based on presence or absence (respectively) of spermatozoa. All listed individuals were mature unless otherwise stated. Age determined by microscopic examination of sectioned sagittal otoliths.

**Fish ID**	**Sample ID**	**Section**	**Rank**	**Species**	**Total Length (mm)**	**Sex determination**	**Sex category for analysis**	**Age determination**	**Age category for analysis**
1	HG1	Hindgut	One	*Centropyge eibli*	91	Male	Male	8	8
10	HG10	Hindgut	One	*Centropyge eibli*	92	Male	Male	11	11
45	HG45	Hindgut	One	*Centropyge eibli*	93	Male	Male	7	7
48	HG48	Hindgut	Three	*Centropyge eibli*	81	Female	Female	3	3
51	HG51	Hindgut	One	*Centropyge eibli*	96	Male	Male	5	5
60	HG60	Hindgut	One	*Centropyge eibli*	93	Male	Male	5	5
76	HG76	Hindgut	Four	*Centropyge eibli*	79	Female	Female	4	4
9	HG9	Hindgut	Five	*Centropyge eibli*	85	Female	Female	5	5
24	HG24	Hindgut	One	*Centropyge eibli X Centropyge flavissima*	87	Male	Male	5	5
28	HG28	Hindgut	One	*Centropyge eibli X Centropyge flavissima*	100	Male	Male	7	7
37	HG37	Hindgut	One	*Centropyge eibli X Centropyge flavissima*	101	Male	Male	8	8
67	HG67	Hindgut	Four	*Centropyge eibli X Centropyge flavissima*	71	Female	Female	2	2
68	HG68	Hindgut	Two	*Centropyge eibli X Centropyge flavissima*	92	Female	Female	3	3
83	HG83	Hindgut	Two	*Centropyge eibli X Centropyge flavissima*	68	Male	Male	3	3
89	HG89	Hindgut	One	*Centropyge eibli X Centropyge flavissima*	106	Male	Male	11	11
95	HG95	Hindgut	Four	*Centropyge eibli X Centropyge flavissima*	76	Female	Female	3	4
101	HG101	Hindgut	One	*Centropyge flavissima*	99	Male	Male	5.5	6
102	HG102	Hindgut	Three	*Centropyge flavissima*	81	Female	Female	3.5	4
104	HG104	Hindgut	One	*Centropyge flavissima*	102	Male	Male	7.5	8
105	HG105	Hindgut	Two	*Centropyge flavissima*	89	Female	Female	4.5	5
106	HG106	Hindgut	Three	*Centropyge flavissima*	84	Female	Female	4.5	5

107	HG107	Hindgut	Four	*Centropyge flavissima*	83	Female	Female	4.5	5
108	HG108	Hindgut	One	*Centropyge flavissima*	92	Male	Male	12.5	13
109	HG109	Hindgut	Two	*Centropyge flavissima*	88	Male	Male	5.5	6
110	HG110	Hindgut	Three	*Centropyge flavissima*	83	Female	Female	3.5	4
111	HG111	Hindgut	Four	*Centropyge flavissima*	76	Female	Female	4.5	5
112	HG112	Hindgut	Five	*Centropyge flavissima*	69	Female	Female	2.5	3
120	HG120	Hindgut	One	*Centropyge flavissima*	104	Male	Male	7.5	8
121	HG121	Hindgut	Three	*Centropyge flavissima*	85	Female	Female	2.5	3
122	HG122	Hindgut	Four	*Centropyge flavissima*	79	Immature female	Female	5.5	6
123	HG123	Hindgut	One	*Centropyge flavissima*	98	Male	Male	10.5	11
124	HG124	Hindgut	Three	*Centropyge flavissima*	88	Female	Female	6.5	7
125	HG125	Hindgut	Four	*Centropyge flavissima*	72	Female	Female	2.5	3
102	MG102	Midgut	Three	*Centropyge flavissima*	81	Female	Female	3.5	4
107	MG107	Midgut	Four	*Centropyge flavissima*	83	Immature female	Female	4.5	5
110	MG110	Midgut	Three	*Centropyge flavissima*	83	Female	Female	3.5	4
120	MG120	Midgut	One	*Centropyge flavissima*	104	Male	Male	7.5	8
122	MG122	Midgut	Four	*Centropyge flavissima*	79	Immature female	Female	5.5	6
123	MG123	Midgut	One	*Centropyge flavissima*	98	Male	Male	10.5	11
124	MG124	Midgut	Three	*Centropyge flavissima*	88	Female	Female	6.5	7

Figure S1: Alpha diversity indices of microbial communities PCR amplified from hindguts and midguts of *Centropyge flavissima* (n=7 each). Boxes denote the interquartile range between the 25th and 75th percentiles of the data, and the horizontal line inside the box reflects the median value. The upper and lower whiskers represent scores outside this middle 50%. Dots outside whiskers represent outliers.

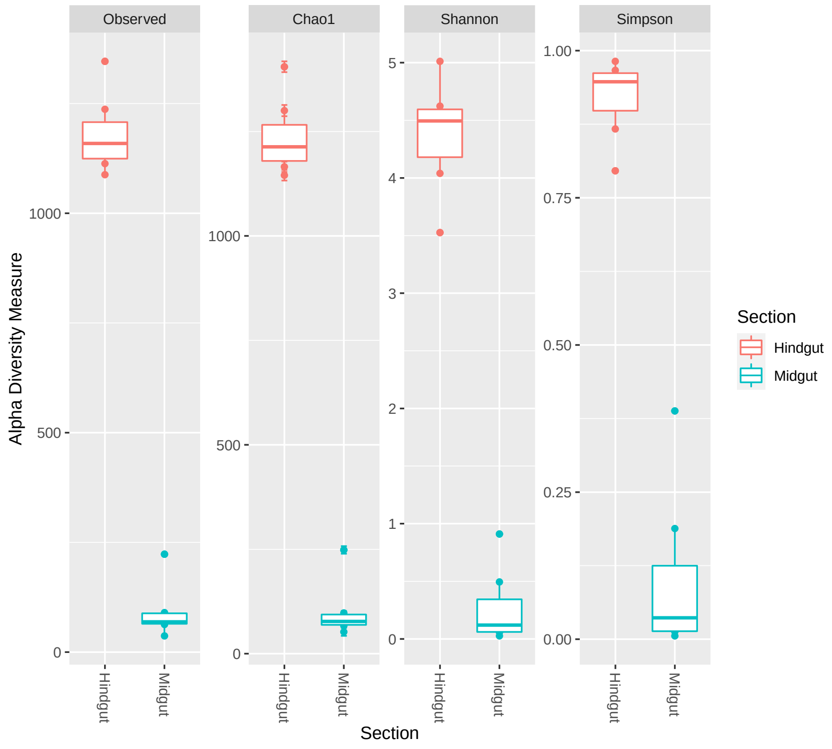

Figure S2: Nonmetric multi-dimensional scaling plot of Bray-Curtis similarity data from hindgut and midgut samples sampled from *Centropyge flavissima* (n=7 of each). Stress =0.051

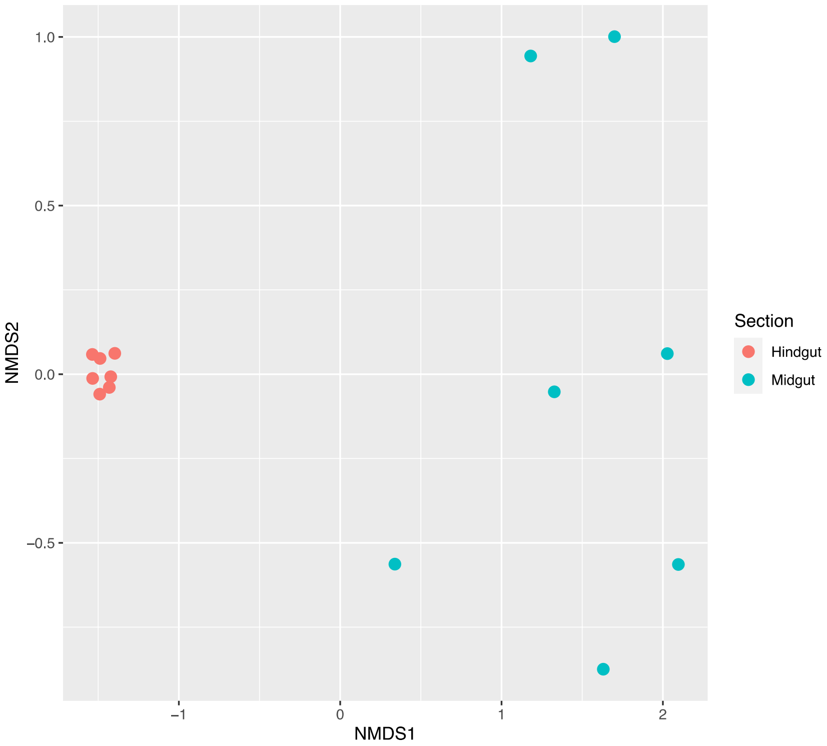

Figure S3: Principal components analysis (PCA) of predicted functional profiles for microbial communities from *Centropyge flavissima* hindgut and midgut samples (n=7) based on PICRUSt2 (Phylogenetic Investigation of Communities by Reconstruction of Unobserved States 2; Douglas et al. 2020).

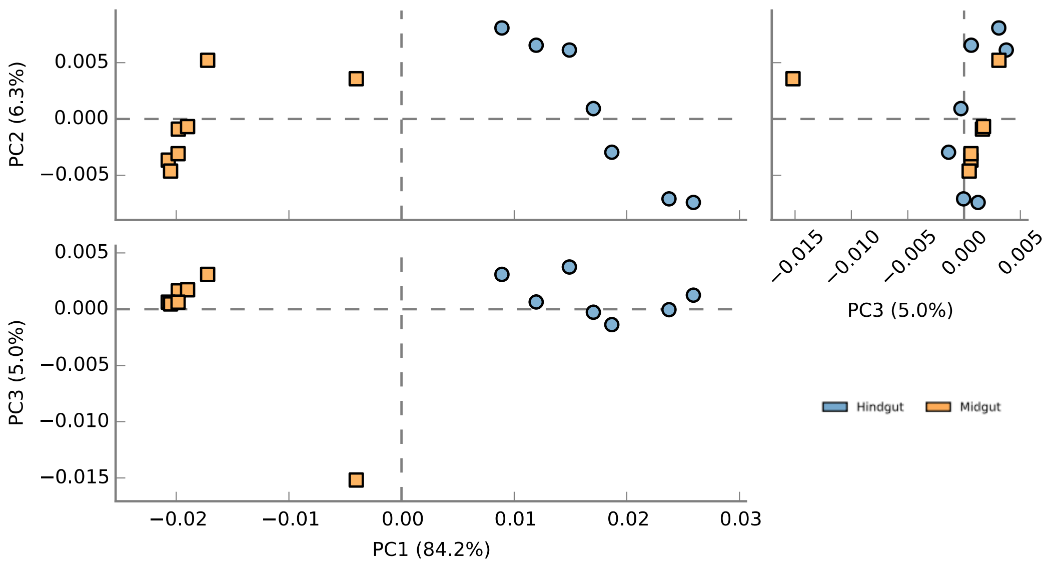

Figure S4: Principal component analysis (PCA) of the predicted functional profiles of hindgut microbial communities from *Centropyge flavissima, C. eibli,* and their hybrids (n=8) based on PICRUSt2 (Phylogenetic Investigation of Communities by Reconstruction of Unobserved States 2, Douglas et al. 2020).

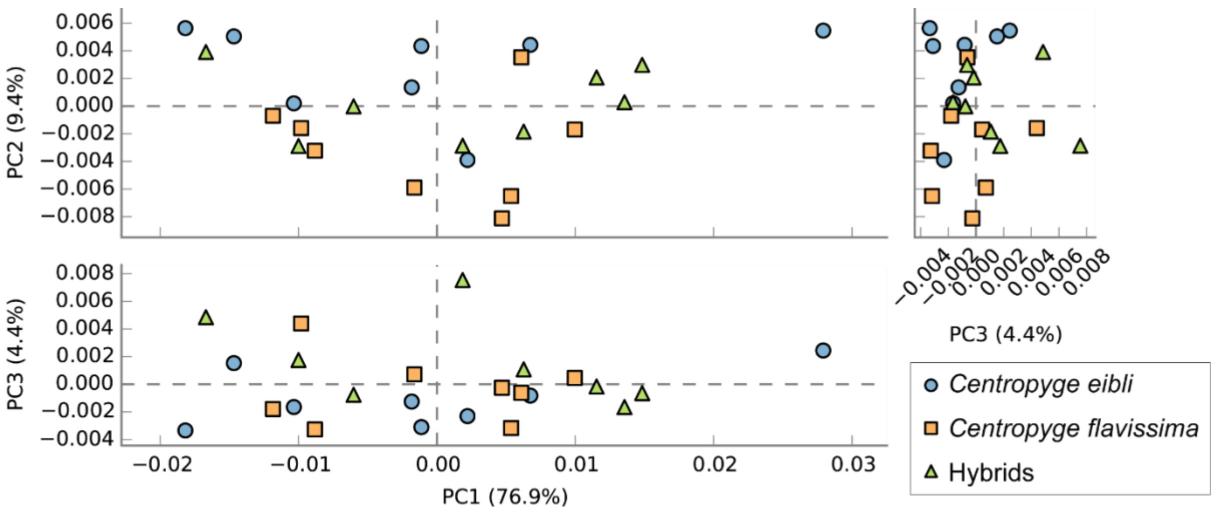

Supplement: Supplementary file 1 — Supplementary Information [file 42003_2023_4919_MOESM1_ESM.pdf]
