# Supplementary material for: Gut microbial communities of hybridising pygmy angelfishes reflect species boundaries
Source: Commun Biol. 2023 May 18;6:542. doi: 10.1038/s42003-023-04919-7 (PMC10195815; doi:10.1038/s42003-023-04919-7)
Supplement: Supplementary file 2 — Description of Additional Supplementary Files [file 42003_2023_4919_MOESM2_ESM.pdf]

Description of Additional Supplementary Files

File name: Supplementary Data 1

Description: OTU table indicating analyses where individual samples were examined

File name: Supplementary Data 2

Description: Significantly differentially abundant predicted metagenomic traits between species (per sample data provided) determined using linear discriminant analysis (LDA) effect size method with a cut off > 2.0 for each pathway
