# Supplementary material for: Gut microbial communities of hybridising pygmy angelfishes reflect species boundaries
Source: Commun Biol. 2023 May 18;6:542. doi: 10.1038/s42003-023-04919-7 (PMC10195815; doi:10.1038/s42003-023-04919-7)
Supplement: Supplementary file 5 — Reporting Summary [file 42003_2023_4919_MOESM5_ESM.pdf]

nature portfolio

Corresponding author(s): Megan Huggett

Last updated by author(s): April 13 2023

Reporting Summary

Nature Portfolio wishes to improve the reproducibility of the work that we publish. This form provides structure for consistency and transparency in reporting. For further information on Nature Portfolio policies, see our Editorial Policies and the Editorial Policy Checklist.

Statistics

For all statistical analyses, confirm that the following items are present in the figure legend, table legend, main text, or Methods section.

n/a	Confirmed	
☐	☒	The exact sample size (*n*) for each experimental group/condition, given as a discrete number and unit of measurement
☐	☒	A statement on whether measurements were taken from distinct samples or whether the same sample was measured repeatedly
☐	☒	The statistical test(s) used AND whether they are one- or two-sided<br>*Only common tests should be described solely by name; describe more complex techniques in the Methods section.*
☐	☒	A description of all covariates tested
☐	☒	A description of any assumptions or corrections, such as tests of normality and adjustment for multiple comparisons
☐	☒	A full description of the statistical parameters including central tendency (e.g. means) or other basic estimates (e.g. regression coefficient) AND variation (e.g. standard deviation) or associated estimates of uncertainty (e.g. confidence intervals)
☐	☒	For null hypothesis testing, the test statistic (e.g. *F*, *t*, *r*) with confidence intervals, effect sizes, degrees of freedom and *P* value noted<br>*Give P values as exact values whenever suitable.*
☒	☐	For Bayesian analysis, information on the choice of priors and Markov chain Monte Carlo settings
☒	☐	For hierarchical and complex designs, identification of the appropriate level for tests and full reporting of outcomes
☐	☒	Estimates of effect sizes (e.g. Cohen's *d*, Pearson's *r*), indicating how they were calculated

Our web collection on statistics for biologists contains articles on many of the points above.

Software and code

Policy information about availability of computer code

Data collection	N/A
Data analysis	These details are provided in the methods section of the manuscript, including the names of software packages used and the specific versions of each.

For manuscripts utilizing custom algorithms or software that are central to the research but not yet described in published literature, software must be made available to editors and reviewers. We strongly encourage code deposition in a community repository (e.g. GitHub). See the Nature Portfolio guidelines for submitting code & software for further information.

Data

Policy information about availability of data

All manuscripts must include a data availability statement. This statement should provide the following information, where applicable:

- Accession codes, unique identifiers, or web links for publicly available datasets
- A description of any restrictions on data availability
- For clinical datasets or third party data, please ensure that the statement adheres to our policy

The genetic data are publicly available in the Sequence Read Archive of the NCBI sequence database under BioProject PRJNA878543 with accession numbers SAMN30732737 to SAMN30732771.

Human research participants

Policy information about studies involving human research participants and Sex and Gender in Research.

Reporting on sex and gender	N/A
Population characteristics	N/A
Recruitment	N/A
Ethics oversight	N/A

Note that full information on the approval of the study protocol must also be provided in the manuscript.

Field-specific reporting

Please select the one below that is the best fit for your research. If you are not sure, read the appropriate sections before making your selection.

☐ Life sciences ☐ Behavioural & social sciences ☒ Ecological, evolutionary & environmental sciences

For a reference copy of the document with all sections, see nature.com/documents/nr-reporting-summary-flat.pdf

Ecological, evolutionary & environmental sciences study design

All studies must disclose on these points even when the disclosure is negative.

Study description	Collection of gut microbial communities from angelfish at Christmas Island in the Eastern Indian Ocean
Research sample	Whole fish specimens were collected by hand spear, immediately placed on ice, and processed within four hours of collection. All procedures were approved by the Animal Ethics Committee at Curtin University (AEC number AEC_2015_25). The entire gastrointestinal (GI) system was dissected out of fishes; individuals with damaged guts were excluded.
Sampling strategy	Samples were taken from the hindgut of 12 C. flavissima individuals and from the midguts of a subset of seven individuals. A further eight C. eibli and hybrids were collected for hindgut analysis. We have previously published studies on microbial communities of fish, seawater, seaweeds, sponges and corals, using as few as n=3 samples, and thus chose to examine a minimum of n=8 samples here.
Data collection	Pygmy angelfish from two species (Centropyge flavissima and C. eibli) and their hybrids were collected in Flying Fish Cove (10° 25'45.7"S , 105°40'05.7"E) at Christmas Island in September 2015, a known hybrid hotspot in the eastern Indian Ocean (Hobbs et al. 2009, 2014). For each individual that we collected, we recorded the location and composition of their harem, sex, and stage of sexual maturity. The mixed species harems that we observed have remained stable (same location on the reef and identical harem members) for between one to six years. Hybrids were identified in situ based on intermediate body colouration
Timing and spatial scale	Samples were collected in Flying Fish Cove (10°25'45.7"S , 105°40'05.7"E) at Christmas Island in September 2015,
Data exclusions	Individuals with damaged guts were excluded
Reproducibility	No laboratory experiments were done in this work.
Randomization	Fish samples for this study were haphazardly selected from the site.
Blinding	Blinding was not relevant to this study as it was an ecological study examining fish in the field.

Did the study involve field work? ☒ Yes ☐ No

Field work, collection and transport

Field conditions	Field conditions were calm, with samples being collected by SCUBA in September 2015.
Location	Pygmy angelfish from two species (Centropyge flavissima and C. eibli) and their hybrids were collected in Flying Fish Cove (10° 25'45.7"S , 105°40'05.7"E) at Christmas Island in September 2015
Access & import/export	All procedures were approved by the Animal Ethics Committee at Curtin University (AEC number AEC_2015_25). Gut material (~1 g)

Access & import/export	was removed and collected into separate, aseptic 80% ethanol suspensions from directly behind the stomach (midgut samples) and from the terminal end of the hindgut (hindgut samples). Samples were immediately frozen at -20 °C for up to 14 days, then stored at -80 °C.
Disturbance	There was minimal disturbance as all samples were collected by experienced divers.

Reporting for specific materials, systems and methods

We require information from authors about some types of materials, experimental systems and methods used in many studies. Here, indicate whether each material, system or method listed is relevant to your study. If you are not sure if a list item applies to your research, read the appropriate section before selecting a response.

Materials & experimental systems

n/a	Involved in the study
☒	☐ Antibodies
☒	☐ Eukaryotic cell lines
☒	☐ Palaeontology and archaeology
☐	☒ Animals and other organisms
☒	☐ Clinical data
☒	☐ Dual use research of concern

Methods

n/a	Involved in the study
☒	☐ ChIP-seq
☒	☐ Flow cytometry
☒	☐ MRI-based neuroimaging

Animals and other research organisms

Policy information about studies involving animals; ARRIVE guidelines recommended for reporting animal research, and Sex and Gender in Research

Laboratory animals	No laboratory animals were used.
Wild animals	Pygmy angelfish from two species (Centropyge flavissima and C. eibli) and their hybrids were collected in Flying Fish Cove (10° 25'45.7"S , 105°40'05.7"E) at Christmas Island in September 2015, a known hybrid hotspot in the eastern Indian Ocean (Hobbs et al. 2009, 2014). For each individual that we collected, we recorded the location and composition of their harem, sex, and stage of sexual maturity. The mixed species harems that we observed have remained stable (same location on the reef and identical harem members) for between one to six years. Hybrids were identified in situ based on intermediate body colouration. Whole fish specimens were collected by hand spear, immediately placed on ice, and processed within four hours of collection. All procedures were approved by the Animal Ethics Committee at Curtin University (AEC number AEC_2015_25).
Reporting on sex	Fish gonads were examined and their condition was recorded upon dissection.
Field-collected samples	Animals were immediately killed and not housed.
Ethics oversight	All procedures were approved by the Animal Ethics Committee at Curtin University (AEC number AEC_2015_25).

Note that full information on the approval of the study protocol must also be provided in the manuscript.
